# Brain-derived neurotrophic factor: a biomarker for obsessive-compulsive disorder?

**DOI:** 10.3389/fnins.2015.00134

**Published:** 2015-04-16

**Authors:** Albino J. Oliveira-Maia, Pedro Castro-Rodrigues

**Affiliations:** ^1^Champalimaud Neuroscience Programme, Champalimaud Centre for the UnknownLisboa, Portugal; ^2^Neuropsychiatry Unit, Champalimaud Clinical Centre, Champalimaud Centre for the UnknownLisboa, Portugal; ^3^Department of Psychiatry and Mental Health, Centro Hospitalar de Lisboa OcidentalLisboa, Portugal; ^4^Centro Hospitalar Psiquiátrico de LisboaLisboa, Portugal

**Keywords:** brain-derived neurotrophic factor (BDNF), obsessive-compulsive disorder (OCD), anxiety disorders, depression, biomarkers

Suliman et al. (2013) have performed a meta-analysis of studies comparing brain-derived neurotrophic factor (BDNF) levels between patients with anxiety disorders and healthy controls. BDNF is initially synthesized as a precursor (pre-pro-BDNF) that is sequentially cleaved into BDNF (Reichardt, 2006). It acts on the TRKB receptor, promoting cellular proliferation, survival and differentiation (Chao, 2003), and is considered to be an important mediator of enduring experience-dependent changes in the brain (Park and Poo, 2013). Furthermore, BDNF and/or other neurotrophic factors may be reduced in disorders such as depression (Duman and Monteggia, 2006), as has been proposed due to the presence of reduced hippocampal volume in depressed patients (Sheline et al., 1996). Thus, there has been much interest in verifying if, across multiple psychiatric disorders, including anxiety disorders, BDNF can be used as a biomarker. Suliman et al. (2013) have synthesized findings in from 8 studies, with a total of 1179 participants, and their findings suggest that BDNF levels are reduced in individuals with anxiety disorders. However, the authors also found that this effect is largely explained by findings in patients with obsessive-compulsive disorder (OCD).

Much of the research regarding a potential role for BDNF in the mechanisms underlying psychiatric disorders has been conducted in the context of animal-models of depressive disorders. Mice with a knock-in of the human loss-of-function Val66Met BDNF gene polymorphism have decreased dendrite length, as well as decreased spine-synapse density, maturity and function in the hippocampus and the prefrontal cortex (Liu and Aghajanian, 2008). This allele is associated with decreased BDNF transport to dendrites and activity dependent release of BDNF, ultimately leading to atrophy of pyramidal neurons in these brain areas (Liu and Zhou, 2012). Furthermore, in tests for depression-like behaviors, such as the learned helplessness or forced swim tests, hippocampal BDNF infusions produce antidepressant-like effects (Shirayama et al., 2002), while targeted hippocampal deletion of BDNF was sufficient to cause depression-like behaviors (Taliaz et al., 2010). There is also evidence that the social defeat stress paradigm leads to downregulation of BDNF transcripts and increases repressive histone methylation (Tsankova et al., 2006). Moreover, BDNF deletion mutants are more vulnerable to stress, as shown by hypothalamo-pituitary axis hyperactivity, impaired working memory and increased depressive-like and anxiety-like behavior (Yu et al., 2012). Finally, mice lacking BDNF have impaired antidepressant responses (Monteggia et al., 2004), and BDNF also seems to be required for the antidepressant actions of fluoxetine on synaptic transmission, long-term potentiation, ocular dominance plasticity and extinction training plasticity (Maya Vetencourt et al., 2008; Karpova et al., 2011; Bath et al., 2012; Duman and Aghajanian, 2012). Ketamine, a new and very promising antidepressant agent, also seems to require BDNF: the synaptogenic actions of this agent are blocked in mice with the Val66Met BDNF gene polymorphism and in BDNF conditional mutant mice (Autry et al., 2011; Liu et al., 2012). However, the view that depression is associated with low levels of BDNF and that BDNF is necessary for antidepressant effects may be too simplistic. In fact, some authors have shown that BDNF has a pro-depressant effect in some brain regions, such as the ventral tegmental area and the nucleus accumbens (Eisch et al., 2003). There is also evidence that male mice with conditional forebrain deletions of BDNF or its receptor do not show depressive-like behavior (Zörner et al., 2003; Monteggia et al., 2007)—arguing for a possible gender-specific role of BDNF in depression (Carbone and Handa, 2013).

The evidence linking BDNF with anxiety-like behaviors in animal models is not as abundant or clear. Foot-shock stress leads to reductions of BDNF levels, arguing for a link between BDNF and anxiety disorders such as post-traumatic stress disorder (Rasmusson et al., 2002). A causal link has been suggested since conditional deletion of BDNF in the postnatal brain leads to hyperactivity after exposure to stressors, and to higher levels of anxiety-like behavior in the light/dark exploration test (Rios et al., 2001). Furthermore, a variant BDNF mouse model (BDNF^Met/Met^), with characteristics that are thought to reproduce the phenotype of humans with the Val66Met polymorphism, has increased anxiety-related behavior in the open field and elevated plus maze (Chen et al., 2006). However, in genetically modified mice overexpressing BDNF in excitatory neurons of the forebrain, including the hippocampus, cortex and amygdala, BDNF overexpression has an unexpected facilitatory effect of anxiety-like behavior in the open field and in the elevated plus maze, concomitant with increased spinogenesis in the basolateral amygdala (Govindarajan et al., 2006). This genetic manipulation also causes antidepressant effects, with improved performance in the forced-swim test and an absence of chronic stress-induced hippocampal atrophy. These findings can be interpreted in light of the contrast between hippocampus and amygdala in depressive and anxiety disorders, with evidence for increased amygdalar volume in anxiety disorders (Anand and Shekhar, 2003) as opposed to decreased hippocampal volume in depressive disorders (Sheline et al., 1996). In any case, this interpretation does not reconcile all of the contradictory findings reported in the literature. Other authors have found that, in rats, a BDNF antisense oligodeoxynucleotide (which reduces BDNF gene expression) provokes anxiety-like behaviors when infused into the central and medial amygdala, but not basolateral amygdala, and that these effects are rescued by BDNF co-infusion (Pandey et al., 2006). Thus, evidence from studies in animal-model studies shows, at best, that the precise relationship between BDNF and anxiety is still unknown.

The meta-analysis by Suliman et al. (2013) raises novel hypotheses for research in the relationship between BDNF and anxiety disorders. The authors found evidence for reduced peripheral BDNF levels in patients with anxiety disorders, mostly due to effects in those suffering from OCD. In fact, in the fifth edition of the Diagnostic and Statistical Manual of Mental Disorders (DSM), OCD has been excluded from the category of anxiety disorders, and rather included in a novel category of obsessive-compulsive and related disorders (American Psychiatric Association, 2013). Several authors had previously suggested a potential role for BDNF in OCD. Hall and colleagues showed a strong association between BDNF gene sequence variants, including the Val66Met variation, and OCD (Hall et al., 2003). Others have confirmed that the Val66Met BDNF gene variant is a risk allele for development of OCD, with possible gender specific effects (Hemmings et al., 2008; Katerberg et al., 2009). However, research exploring a possible pathophysiological role for BDNF in OCD is lacking. The work by Suliman et al. (2013) summarizes research that suggests lower BDNF levels in patients with OCD, raising interesting possibilities for clinical research, such as the possibility of testing peripheral BDNF as a biomarker for this disorder. Furthermore, it also reinforces the need for further research to clarify a potential role for BDNF in the neurobiology of OCD.

## Conflict of interest statement

The authors declare that the research was conducted in the absence of any commercial or financial relationships that could be construed as a potential conflict of interest.
